# Health and Environmental Risks from Lead-based Ammunition: Science Versus Socio-Politics

**DOI:** 10.1007/s10393-016-1177-x

**Published:** 2016-09-23

**Authors:** Jon M. Arnemo, Oddgeir Andersen, Sigbjørn Stokke, Vernon G. Thomas, Oliver Krone, Deborah J. Pain, Rafael Mateo

**Affiliations:** 1 Department of Forestry and Wildlife Management, Hedmark University College, Campus Evenstad, 2480 Koppang, Norway; 2 Department of Wildlife, Fish, and Environmental Studies, Swedish University of Agricultural Sciences, Umeå, Sweden; 3 Norwegian Institute for Nature Research, Lillehammer, Norway; 4 Norwegian Institute for Nature Research, Trondheim, Norway; 5 Department of Integrative Biology, College of Biological Science, University of Guelph, Guelph, ON Canada; 6 Department of Wildlife Diseases, Leibniz Institute for Zoo and Wildlife Research, Berlin, Germany; 7 Wildfowl & Wetlands Trust, Slimbridge, Gloucestershire UK; 8 Institute of Research in Game Resources, IREC (CSIC-UCLM-JCCM), Ciudad Real, Spain

**Fig. 1:**
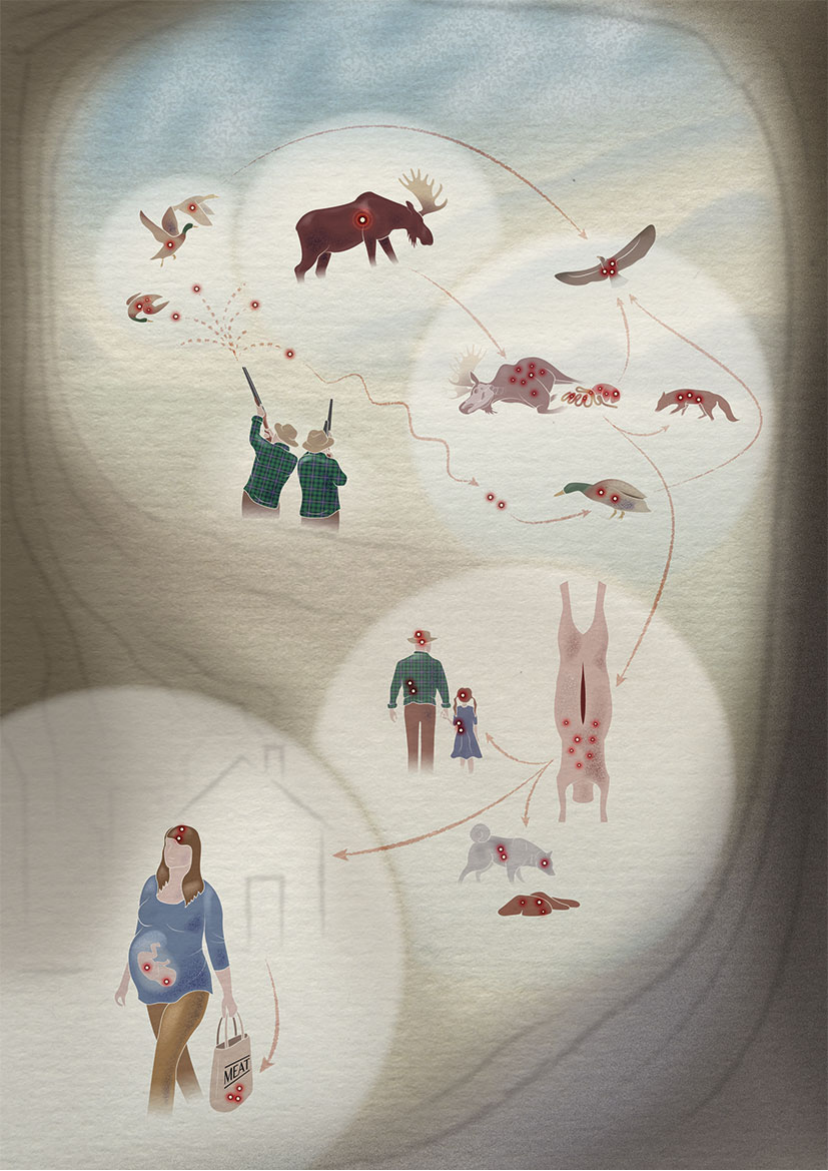
The cycle of lead from spent ammunition. Lead from gunshot or bullets will enter the food chain and expose humans and animals to health risks [© Diogo Guerra 2015]

Lead (Pb) is toxic and is banned from gasoline, paints, and various household items in most developed countries. Lead ammunition, however, is still widely used for hunting and shooting, and is now likely the greatest, largely unregulated source of lead that is knowingly discharged into the environment in the USA (Health Risks from Lead-Based Ammunition in the Environment—A Consensus Statement of Scientists 2013; U.S. Geological Survey 2013). For decades, poisoning from spent lead ammunition was mainly regarded as a disease of waterfowl (Bellrose 1959), but it also puts at risk the health of raptors, scavengers, and other terrestrial species, including humans who frequently consume hunted game (Fig. 1). Scientists across North America and Europe have published consensus statements on the risks to wildlife, the environment and human health from the use of lead ammunition, and the need for its replacement by non-toxic alternatives (Health Risks from Lead-Based Ammunition in the Environment—A Consensus Statement of Scientists, 2013; Group of Scientists 2014). This is now a pressing *One Health* issue.

We carried out a literature search in the database Web of Science for scientific papers dealing with environmental and health consequences of the use of lead in ammunition. We used 11 different query combinations of the key words “lead, lead-free, non-lead, non-toxic, ammunition, hunting, poisoning, shot, meat, game, raptor, waterfowl, and upland game.” After removing non-relevant papers, we manually added approximately 100 references found by searching in other databases (PubMed, Google Scholar) or in reference lists of published literature. Finally, we were left with 570 peer-reviewed papers published from 1975 through August 2016. The number of articles per year showed a strong increase over time during the period covered: 6.9 in 1975–1989, 9.3 in 1990–1999, 19.0 in 2000–2009, and 27.7 in 2010–2016. These papers were analyzed for relevance and conclusions, with special reference to topics such as health risks for humans consuming game hunted with lead-based ammunition (e.g., Johansen et al. 2004), lead residues in game meat intended for human consumption (e.g., Andreotti et al. 2016), use of apex species as biomonitoring sentinels for lead exposure and effects (e.g., Mateo-Tomás et al. 2016), lead poisoning of critically endangered species (e.g., Bakker et al. 2016), scavengers (Bedrosian et al. 2012), upland game birds (e.g., Kreager et al. 2008), and waterfowl (e.g., Green and Pain 2016), lead fragments in carcasses and offal (Cruz-Martinez et al. 2015), and lead contamination from shooting ranges (Okkenhaug et al. 2016). We found that more than 99% of them raised concerns over use of lead-based ammunition. A recent international symposium (Delahay and Spray 2015) highlighted the health and environmental risks from lead in spent ammunition. Apparently, there is scientific consensus on these issues.

According to the World Health Organization (WHO 2015) and the European Food Safety Authority (EFSA 2013), there are no defined safe levels of lead intake in humans. The toxic effects of lead are numerous and largely irreversible. Of greatest concern is the effect on the nervous system of fetuses and children. The adverse effect of lead on children’s intellectual function is well established, especially the decline in IQ and loss of cognitive skills (Lanphear et al. 2005; Bellinger 2008; Grandjean and Landrigan 2014), which may have huge economic effects on societies especially when populations are affected. There is a strong positive relationship between childhood lead exposure and subsequent aggressive crime (Taylor et al. 2016). Additionally, Menke et al. (2006) showed that increased cardiovascular mortality in adults occurs at substantially lower blood lead levels than previously reported. Despite the marked decrease in blood lead levels in the general population, low-level environmental lead exposure remains a major public health problem and has been termed a “silent killer” (Nawrot and Staessen 2006). People who frequently consume game shot with lead ammunition are at risk from high dietary lead exposure, e.g., Greenlanders had mean blood lead levels four to ten times higher than the EFSA benchmark dose modeling (BMDL) thresholds for developmental neurotoxicity in children and for chronic kidney disease in adults (Johansen et al. 2006). Bjermo et al. (2013) showed that increased blood lead levels in Swedish adults were associated with wild game consumption and that the blood lead concentrations in several individuals exceeded EFSA’s BMDL threshold values. The sources of lead in wild game were hunting bullets or shot.

Lead-based bullets fragment inside game animals, potentially contaminating much of the carcass (Hunt et al. 2009). This explains high concentrations of lead found in retailed packages of venison. The mean lead concentration in packages of ground meat from Norwegian moose killed with lead-based bullets was 56 times the European Commission maximum level (ECML) for lead in other meat (Lindboe et al. 2012). Similar reports exist for game meat sold in the UK, with mean levels in game birds prepared for the table, following the removal of shot and visible large fragments, being 12 times the ECML (Pain et al. 2010; Green and Pain 2015). A recent report from Canada (Fachehoun et al. 2015) recommended that vulnerable groups and individuals who consume venison on a weekly basis should avoid meat from animals killed with lead ammunition. The lead in particles of ingested ammunition fragments can be transformed to soluble lead ions and absorbed (Barltrop and Meek 1979), and cooking in acidic media may increase its bioavailability in humans (Mateo et al. 2011). A number of European food safety agencies now advise children and women of pregnancy age to avoid eating game shot with lead (Knutsen et al. 2015).

While chronic exposure to lead predisposes humans to numerous disease conditions (Meyer et al. 2008), it kills many waterbirds and terrestrial birds that ingest lead from spent ammunition (Pain et al. 2009, 2015). Sub-lethal effects of lead on immune function and reproduction of birds can also have negative consequences (Vallverdú-Coll et al. 2015). A literature review revealed that globally, in addition to wildfowl, 33 raptor species and 30 other terrestrial bird species were reported to have ingested and/or been poisoned by lead fragments from hunters’ ammunition (Pain et al. 2009). Also, Johnson et al. (2013) reported that lead contamination of carcasses remains a serious threat to the health and sustainability of scavenging birds. To reduce significant poisoning of migratory birds, in November 2014, contracting parties to the Convention on Migratory Species (CMS 2014) adopted a resolution that includes a 2017 deadline for the phase out of all lead ammunition in both terrestrial and wetland habitats.

Legislative actions to reduce the risks from lead ammunition have been taken in some places, primarily requiring the use of non-toxic gunshot for shooting wildfowl or over wetlands (Mateo 2009). In the USA, where non-toxic shot has been required nation-wide for waterfowl hunting since 1991, waterfowl mortality from lead shot ingestion is considered to have declined considerably (Anderson et al. 2000; Samuel and Bowers 2000). According to Kanstrup (2015), the Danish prohibition of ownership and use of lead shot has not had a long-term detrimental effect on participation in hunting and the numbers of animals taken with non-toxic shot substitutes. In other countries, restrictions have been ineffective, due to poor compliance, lack of enforcement, and their partial nature (Cromie et al. 2015; Green and Pain 2016). Few measures have been taken to reduce mortality in terrestrial birds, including raptors that ingest lead from gunshot and bullets while feeding on shot prey, although progress is being made in a few places (Krone et al. 2009). Overall, national responses to this problem have been incomplete, piecemeal, and widely opposed by some shooting stakeholder groups.

In *Merchants of Doubt*, Oreskes and Conway (2010) showed how denial of scientific evidence has been a strategy used by those with vested interests in important health and environmental issues such as climate change, tobacco smoking, ozone layer thinning, acid rain, and DDT. The ongoing discussion on the use of lead-based ammunition parallels the debates covered by Oreskes and Conway. Thus, extensive scientific evidence is disputed or rejected, lead substitutes developed by the ammunition industries are deemed inadequate or too expensive, and proposed bans on lead ammunition are often viewed as anti-hunting. Less than 5% of the Europeans are sport hunters. Their political impact, however, is disproportionately large. Hunters are well-organized at national and international levels, and are represented effectively by industry and wealthy politically influential groups, including heads of state and royalty. Despite the global scientific consensus on the health and environmental risks from lead exposure, major lobbyist organizations are opposing any attempt to ban, or even restrict, the use of lead-based ammunition. While certain shooting umbrella organizations acknowledge the problem, they have shown little leadership in tackling it.

Recent events in the USA and Europe illustrate these points. California passed the Assembly Bill 711 in 2013, which will ban the use of lead ammunition for all hunting from 2019. This is the first jurisdiction to protect humans and wild animals from lead exposure from all spent ammunition. The US National Rifle Association (NRA 2013), rejecting the scientific evidence showing the danger of lead exposure in humans and animals, strongly opposed the California decision and declared it an “anti-hunting bill.”

The reticence of the shooting community exists, also, in Norway. In February 2015, after a decade of lobbying by shooting interests, the Norwegian Parliament rescinded the total ban on lead shot used for hunting outside wetlands. An overwhelming majority of MPs (79 against 16) supported the bill, disregarding scientific evidence on health and environmental risks from lead-based ammunition. The Norwegian Association for Hunters and Anglers (NJFF 2015) described the reintroduction of lead shot for hunting as a “victory,” and the Association of European Manufacturers of Sporting Ammunition (AFEMS 2015) called the decision a “great success.”

The AFEMS (2015) promotes the use of lead-based ammunition on their website stating that “All the European industries using metallic lead, including ours, are acting for countering the classification of metallic lead as a toxic substance.” This decision contravenes every scientific study on the toxicity of ingested lead. In a press release from a recent symposium, AFEMS and The World Forum on Shooting Activities (WFSA) state that: “… metallic lead in ammunition has no significant impact on human health and the environment as compared to other forms of lead. Lead fragments in game meat, if ingested, cannot be directly absorbed by the human body because they are in metallic form” (AFEMS/WFSW 2015). These conclusions repudiate the huge body of research demonstrating the toxic consequences of lead shot and bullet fragment ingestion, ignoring that ingested metallic lead is solubilized then absorbed and exerts its toxic effects on the entire body.

Despite overwhelming scientific evidence and increasing policy imperatives, nationally regulated bans on the use of lead shotgun and rifle ammunition are few. North American and European arms industries have developed non-toxic shot and bullets that are as effective and comparably priced as their lead counterparts (Thomas 2015). Our understanding of the deleterious impacts of this form of lead exposure on wildlife and humans will change little with further scientific research, no more evidence is required. The same rationales that were used to remove lead from gasoline, paints, and household items should be applied to lead-based hunting ammunition, nationally and internationally. This is now a socio-political issue.
